# The effects of sign language on spoken language acquisition in children with hearing loss: a systematic review protocol

**DOI:** 10.1186/2046-4053-2-108

**Published:** 2013-12-06

**Authors:** Elizabeth M Fitzpatrick, Adrienne Stevens, Chantelle Garritty, David Moher

**Affiliations:** 1 Audiology and Speech-Language Pathology Program, Faculty of Health Sciences, University of Ottawa, 451 Smyth Road, Ottawa, ON K1H 8M5, Canada; 2 Children’s Hospital of Eastern Ontario Research Institute, 401 Smyth Road, Ottawa, ON K1H 8LM, Canada; 3 Centre for Practice-Changing Research, Ottawa Hospital Research Institute, 501 Smyth Road, Box 201B, Ottawa, ON K1H 8L6, Canada; 4 Department of Epidemiology and Community Medicine, Faculty of Medicine, University of Ottawa, 501 Smyth Road, Box 201B, Ottawa, ON K1H 8L6, Canada

**Keywords:** Children, Hearing loss, Spoken language, Sign language, Outcomes, Systematic review

## Abstract

**Background:**

Permanent childhood hearing loss affects 1 to 3 per 1000 children and frequently disrupts typical spoken language acquisition. Early identification of hearing loss through universal newborn hearing screening and the use of new hearing technologies including cochlear implants make spoken language an option for most children. However, there is no consensus on what constitutes optimal interventions for children when spoken language is the desired outcome. Intervention and educational approaches ranging from oral language only to oral language combined with various forms of sign language have evolved. Parents are therefore faced with important decisions in the first months of their child’s life.

**Methods/Design:**

This article presents the protocol for a systematic review of the effects of using sign language in combination with oral language intervention on spoken language acquisition. Studies addressing early intervention will be selected in which therapy involving oral language intervention and any form of sign language or sign support is used. Comparison groups will include children in early oral language intervention programs without sign support. The primary outcomes of interest to be examined include all measures of auditory, vocabulary, language, speech production, and speech intelligibility skills. We will include randomized controlled trials, controlled clinical trials, and other quasi-experimental designs that include comparator groups as well as prospective and retrospective cohort studies. Case-control, cross-sectional, case series, and case studies will be excluded. Several electronic databases will be searched (for example, MEDLINE, EMBASE, CINAHL, PsycINFO) as well as grey literature and key websites. We anticipate that a narrative synthesis of the evidence will be required. We will carry out meta-analysis for outcomes if clinical similarity, quantity and quality permit quantitative pooling of data. We will conduct subgroup analyses if possible according to severity/type of hearing disorder, age of identification, and type of hearing technology.

**Discussion:**

This review will provide evidence on the effectiveness of using sign language in combination with oral language therapies for developing spoken language in children with hearing loss who are identified at a young age. The information from this review can provide guidance to parents and intervention specialists, inform policy decisions and provide directions for future research.

**PROSPERO registration number:**

CRD42013005426

## Background

Population-based newborn hearing screening has become a standard of care in the developed world to improve the developmental outcomes for children with permanent hearing loss [1-3]. Childhood hearing loss is one of the most common congenital disorders affecting 1 to 3 per 1000 live births [3-5] resulting in 1,000 to 1,200 affected children born in Canada annually [6]. Hearing loss interferes with typical language acquisition and children are at risk of developing sub-optimal spoken language, and consequently poorer academic and literacy skills, than their peers with normal hearing [7-10]. While there is good evidence that newborn screening achieves early identification of hearing loss, the evidence for the effectiveness of screening in improving spoken language outcomes has been less convincing [2,11-13]. It is well recognized that early identification is not sufficient to improve communication skills and that intervention using hearing prostheses and early language stimulation must follow [1,14].

Historically, there has been a debate about what constitutes desirable outcomes for children with hearing loss and a plethora of intervention/education programs have evolved. These programs can be grouped into two distinct philosophies: one focused on fostering spoken language development and inclusion with normal hearing peers; and the other on communication using primarily a visual approach (for example, sign language) and promoting a sense of identity with a Deaf culture [15,16]. Epidemiologic data confirm that more than 90% of children with hearing impairment are born to families where both parents have normal hearing [17]. Although there is generally a consensus that various treatment options with different outcome goals should be available to children and families, data from the Ministry of Child and Youth Services in Ontario (personal communication), where the first province-wide universal screening program was implemented in 2002, indicate that more than 90% of families choose a spoken language option for their children. This review will therefore address the issue of interventions aimed at developing spoken language outcomes and will not deal with philosophical differences that exist between various stakeholders involved in supporting children with hearing loss and their families.

Despite a growing body of evidence that children with hearing loss can develop oral language skills [9,18,19], there have been longstanding disagreements about optimal interventions when spoken language development is the desired outcome [20]. Although there is substantial and variable anecdotal evidence supporting various intervention approaches, there is little scientifically based consensus. However, this information is necessary to: 1) guide families in making decisions about care for their children in infancy; 2) inform clinicians so that they can design and tailor treatment plans to achieve the desired outcomes; and 3) inform policy makers so they can make decisions about investments in early intervention services.

From a theoretical perspective, research in neuroplasticity and critical periods of learning [21,22] lend support to the theory that optimal intervention involving rich auditory and oral language stimulation should be initiated in infancy to mitigate the effects of hearing loss [23-25]. The expectation then is that oral language outcomes of children receiving auditory-based intervention should be better than those of children who are deprived of this intensive auditory stimulation through the addition of visual information which might slow down or interfere with spoken language acquisition. Another body of research suggests that signed languages such as American Sign Language (ASL) are processed in the brain in the same manner as spoken languages, are therefore complementary, and provide a strong foundation for learning oral languages [26,27]. Applying this reasoning, associations such as the Bilingual Coalition of Canada have called for access to ASL for all children with hearing loss even when parents choose spoken language as the desired mode of communication for their child.

Further complicating the issue for parents is the growth of the use of signs, commonly known as “baby sign language” with typically developing babies. This practice raises further questions about whether the addition of signs might promote spoken language acquisition in children with impaired hearing. A systematic review [28] found no evidence supporting the use of baby signs or any indication that exposure to baby signs interfered with children’s typical language acquisition. Recently, the American Academy of Pediatrics concluded that baby sign language is a tool for improving communication in young children with normal hearing [29].

The uncertainty surrounding the most appropriate interventions required to achieve age-appropriate language skills is one of the major challenges facing newborn hearing screening initiatives. This review is timely because there has been a proliferation of newborn hearing screening programs worldwide in the past 10 years. In addition to early identification, early cochlear implantation “which involves electrical stimulation of the auditory system” has had a dramatic impact on improving the spoken language potential for children with severe to profound deafness. There is good evidence for positive spoken language outcomes from the cochlear implant literature [18,25,30] but considerable debate continues about what constitutes optimal intervention approaches for promoting spoken language acquisition, regardless of degree of hearing loss. In essence, the new possibilities for children due to newborn hearing screening, new technologies, and emerging evidence on using signs with normal hearing children have re-ignited the debate on best practices for children with hearing loss. Parents are therefore faced with important decisions in the early months of their child’s life when they first learn of the presence of permanent hearing loss.

Accordingly, the primary purpose of this research is to connect researchers and knowledge users to examine the evidence for the effects of various treatment options for early-identified children with hearing loss when the desired outcome is spoken communication. The major question addressed in this proposal is specific to the components or characteristics of the intervention and the auditory and spoken language capacity of the developing child. A review undertaken in 2008 to update the United States Preventive Services Task Force recommendations on newborn hearing screening highlighted the need for further research to demonstrate the effectiveness of the entire process of screening to intervention [2].

In 2002, as Ontario was implementing newborn hearing screening, a review commissioned by Health Canada [31] compared outcomes for various interventions across the spectrum from oral to sign language approaches but failed to find conclusive evidence, citing study quality as an important barrier to knowledge synthesis in the field. Since this 10-year old Health Canada review, technology has dramatically impacted pediatric hearing care such that early intervention for all children and early cochlear implantation (including recently the use of bilateral implants) to treat the most severe losses have become the new standards of care. Several new studies investigating the impact of newborn hearing screening outcomes and of early cochlear implantation have been published since the 2002 review.

### Objectives of the review

The objective of this systematic review is to answer the following question: do children with hearing loss have better spoken language outcomes when exposed to early intervention that uses signs to support language compared with language intervention without sign language? In addition, potential moderating factors that may impact spoken language development will be investigated, if possible.

## Methods/Design

### Description

This protocol is reported according to Preferred Reporting Items for Systematic Reviews and Meta-Analyses for Protocols (PRISMA-P) [32] and uses an integrated knowledge translation approach throughout the conduct of the systematic review. Each component of the project includes stakeholders to ensure relevance of the project for parents, clinicians, and decision-makers. The protocol has been registered in PROSPERO (Registration # CRD42013005426), an international register of prospective systematic review protocols.

### Search methods for study identification

A comprehensive search strategy was developed by an experienced information specialist and peer reviewed using the Peer Review of Electronic Strategies tool [33]. The electronic databases MEDLINE, EMBASE, CINAHL, PsycINFO, and the Cochrane CENTRAL database of controlled trials will be searched. A search strategy developed for MEDLINE is shown in Additional file 1 and will be adapted as required for each database.

‘Grey literature’ searches will be conducted for other potentially relevant articles. We will hand search recently published issues of key journals (for example, *Ear and Hearing*, *Journal of Deaf Studies and Deaf Education*) for additional studies not yet indexed in electronic databases. Proceedings and abstracts from key conferences (Newborn Hearing Screening Conference, International Cochlear Implant Symposium in Children, and A.G. Bell Conference) as well as relevant professional websites (A.G. Bell Association for the Deaf, National Acoustics Lab, National Health Services (United Kingdom) Newborn Hearing Screening Programme) will also be searched to identify unpublished studies.

### Selection criteria

#### Study designs

It is anticipated that this review will include primarily non-randomized studies as well as observational studies as our previous review in this field of study yielded only one randomized controlled trial [31]. Accordingly, we will include the following study designs: randomized clinical trials, controlled clinical trials and other quasi-experimental designs that include comparator groups, prospective cohort studies, and retrospective cohort studies. Case-control, cross-sectional, case series, and case studies will be excluded.

#### Population

Included studies will meet the following criteria: 1) children with permanent hearing loss of any degree of severity and of early onset (prior to age 2 years) and typically using hearing aids or cochlear implants; and 2) children identified and enrolled in early intervention programs by 2 years of age.

Studies that include only outcomes of children with developmental disabilities that involve cognitive delay in addition to hearing impairment will be excluded.

#### Intervention

We will include studies addressing early intervention aimed at spoken language development, at least comprised of: 1) therapy involving oral language stimulation; and 2) therapy including any form of sign language or sign support (for example, Signing Exact English, ASL, Langue des signes québeçoise, sign assist, baby sign language), which may form part of an intervention program (for example, total communication, dual communication, simultaneous communication, bilingual approaches).

Relevancy of papers will be assessed on the basis of the components of the approach (that is, spoken language intervention with or without some form of sign language included), and not on the basis of the program label.

#### Control/comparison

Control groups will include children receiving early intervention spoken language therapy without sign language (that is, hearing technology and oral language stimulation).

### Outcomes

#### Primary outcomes

Primary outcomes will include all measures of listening and spoken language development including auditory skills (for example, speech perception tests), oral receptive and expressive vocabulary and language, speech production, and speech intelligibility. These outcomes are well supported in the recent literature as clinically relevant outcomes [13,18,25]. However, during the broad and focused screening stages, articles will not be excluded on the basis of outcomes.

#### Secondary outcomes

Electrophysiologic outcomes (for example, auditory brainstem or cortical responses) will also be examined to document potential “objective” benefits of various types of intervention.

#### Adverse outcomes

Any adverse outcomes as reported in studies will be collected.

### Time frame

Given our interest on the effects of visual (sign) languages on early identified children, we will only include studies published 1995 onward as previous generations of children were unlikely to receive the same standards of care related to early identification of hearing disorders and access to new hearing technologies (for example, cochlear implantation).

### Language

For feasibility, only articles written in the English and French languages will be included. Articles in other languages will be screened at the broad screening level for potential relevance and the details of any relevant citations will be included in the final report.

### Data collection

#### Study selection

Once all records have been retrieved through electronic and other searching methods, they will be compiled in a Reference Manager database and checked for duplication. All remaining citations will then be exported to the Distiller Systematic Review Software (DSR), an internet-based software program [http://systematic-review.net/] for the study selection process.

The study selection involves two specific stages. Screening forms will be developed from the inclusion and exclusion criteria and calibrated among reviewers with a subset of records before each screening stage takes place.

#### Broad screening

Titles and abstracts will be assessed by one reviewer for potential relevance; a second reviewer will verify those records deemed not relevant.

#### Focused screening

Two independent reviewers will screen all potentially relevant full-text articles. Disagreements will be resolved by consensus or by consultation with a third member of the research team when needed.

### Data abstraction

Electronic study-specific data abstraction forms will be used to abstract pre-determined data for each study. Data will be collected and managed in DSR. Data abstraction items will broadly include: 1) study characteristics (author names and contact information, year, institution, country, language, publication status, source of funding); 2) study design; 3) population characteristics - for example, sample size, sex, ethnicity, etiology (including radiologic findings when available), age of hearing loss identification and intervention, severity of hearing loss, type of hearing technology, time with hearing technology, parental involvement, socio-economic status, home/intervention language, cognitive status; 4) details of intervention (including fidelity); 5) details of control or comparison groups (including fidelity); 6) risk of bias assessments; and 7) outcome definitions and data. The data abstraction form was finalized with input from the knowledge users at the first team meeting. One researcher will extract all information; a second reviewer will verify all information. Discrepant findings will be resolved through consensus or a third reviewer when required.

### Missing data

If information or data are missing or incomplete, we will attempt to contact the study authors twice through email over a 4-week period. We will not impute data for any outcomes.

### Risk of bias assessment

The risk of bias assessment will be conducted by one researcher with full verification by a second researcher. For randomized and controlled clinical trials, the Cochrane Risk of Bias tool [34] will be used; domains of assessment relate to selection, performance, attrition, detection, selective reporting, and other biases. For quasi-experimental studies (interrupted time series and controlled before-after designs) we will use the Effective Practice and Organisation of Care modification of the Cochrane Risk of Bias tool. Cohort designs or case controlled studies will be assessed using the Qualitative Assessment Tool for Quantitative Studies, a tool developed by the Effective Public Health Practice Project at McMaster University to assess the quality of studies in a systematic review [35,36]. The tool, accompanied by a reviewer's dictionary, results in an overall methodological rating of studies based on an appraisal of eight areas: selection bias, study design, confounders, blinding, data collection methods, withdrawals and dropouts, integrity of intervention and study analysis. Any disagreements in assessments will be resolved through discussion or by third party adjudication.

### Data analysis

Study characteristics will be summarized narratively in the text and/or in tables in the report; data may be presented as frequencies and percentages, medians and interquartile ranges, or means and standard deviations, where appropriate. A narrative synthesis of the evidence will be conducted when quantitative pooling of data is not possible. Based on previous reviews [31,37,38] that showed great heterogeneity in research designs and wide variability in methods and types of spoken language outcomes, it is anticipated that a full meta-analysis will not be possible.

If, based on clinical similarity, quantity, and quality, meta-analysis is possible for some outcomes, we will use a random effects model to generate aggregate results. For continuous data we will compute mean or standardized mean differences with 95% confidence intervals (CIs). Any change-from-baseline data will be collected in addition to final data. Risk ratios with 95% CIs will be computed for dichotomous data. Decisions about handling and analyzing ordinal outcomes will be determined *post hoc*, subject to the body of evidence available. For any time-to-event data, the generic inverse variance method will be used to meta-analyze outcomes using log hazard ratios and standard errors. When required, we will convert data (for example, from standard error to standard deviation) to facilitate analyses and consistency in the presentation of study findings. Statistical heterogeneity will be evaluated using I-squared (I^2^) statistics; for the interpretation of I^2^, a rough guide of low (0-25%), moderate (25-50%), substantial (50-75%), and considerable (75-100%) heterogeneity will be used; possible reasons contributing to heterogeneity will be explored. If there is considerable heterogeneity (>75%), we will not complete a pooled analysis. If data permit, sensitivity analyses may be undertaken with respect to risk of bias (restricting to studies with low risk of bias), the fidelity of the intervention, data issues, or measurement of outcomes. If at least 10 studies are included in a meta-analysis, funnel plots will be generated to assess for publication bias and other possible reasons for asymmetry [39].

### Subgroup analyses

We will examine the following variables in subgroup analyses as effect modifiers, if feasible: 1) severity of hearing loss (mild, moderate, severe); 2) children with auditory neuropathy; 3) age of identification of hearing loss (<6 months, 6 to 12 months, >12 months); and 4) children with hearing technology (cochlear implants, hearing aids).

If appropriate and with sufficient, complete data, we will verify results of the subgroup analyses with univariate meta-regression. The variables outlined above for subgroup analyses will be considered statistically significant at *P* < 0.01.

### Grading the strength of evidence

We will apply methodology developed by the Grades of Recommendation Assessment, Development and Evaluation working group [40] to rate the evidence for primary and adverse outcomes. The body of evidence for each outcome will be assessed across the domains of risk bias, consistency, directness, precision, and publication bias. The quality of the evidence will be rated as high (very confident that true effect is close to the estimate of the effect), moderate (moderately confident in the effect estimate), low (limited confidence in the effect estimate), or very low (little confidence in the effect estimate). We will discuss the results in light of the strength of findings as well their research and potential practice and family counseling implications.

### Reporting

Reporting of the review findings will follow guidelines for reporting systematic reviews presented in the PRISMA statement [32]. The report will include a flow chart detailing the reasons for included and excluded studies. Guided by knowledge user input, the review findings will subsequently be translated in formats that are adapted to different audiences including clinicians and parents.

### Engagement of knowledge users

The principal objective of this review is to understand the effects of using sign language as a support for spoken communication development of children with hearing loss. This project represents collaboration between researchers and three knowledge users representing parents, health and education provider groups with a broad reach, who bring complementary but distinct expertise and strengths that will facilitate knowledge translation. Intervention decisions and parent guidance tend to be based on single studies without attention paid to the quality of the evidence and the particular characteristics of the study population. The expected outcome of partnering closely with end-users is to eliminate some of the potential pitfalls in the way that evidence is interpreted and used. We envision that knowledge translation will take place continually through natural networks and outreach activities, as knowledge users familiarize themselves with the relevant literature and the results of this systematic review. Planned activities include in-service professional meetings, a parent conference presentation, parent information pamphlet, presentation at scientific conferences, peer-reviewed publication, and the integration of the results into academic seminars and coursework.

## Discussion

Infant hearing screening continues to occupy an important place on national and international agendas as a population health intervention aimed at improving communication development for children with permanent hearing disorders. Hearing screening has received widespread support on the basis of the burden of the disorder to society and the assumption that early intervention can prevent or reduce delays in language development. However, there remains considerable uncertainty and disagreement about what constitutes optimal treatments for the current generation of early-identified children with hearing loss. Successful implementation of evidence into the pediatric hearing field has been difficult to achieve with the end result that unbiased information is not readily available to parents. Consequently, parents are forced to make decisions about care based on unclear and potentially biased evidence when their children are diagnosed in infancy.

This systematic review aims to become an important information tool for parents, clinicians, researchers, and decision-makers who need new evidence that is accumulating in the field of newborn screening and early intervention outcomes. Our goal is that this review will contribute to a better understanding of treatment effects, leading potentially to a timelier introduction of the most effective interventions that are aligned with parents’ desired outcomes for their children with hearing loss. Through a knowledge-user and researcher partnership, we will create a common understanding of the goals, use a common terminology, and assemble the best possible evidence to provide care to the “new” populations of children with early-identified hearing loss.

This review was motivated by questions and concerns at the clinical practice level of providing services for young children. One potential byproduct of this review is the development of clear research needs for the scientific community grounded in the experiences of the knowledge users. In the long-term, we envision that the findings resulting from this collaboration that spans healthcare, education, and graduate training will help to further define appropriate questions for future collaborative research to address childhood hearing loss issues in a field wrought with anecdotal reports and expert opinion. Accordingly, this review is expected to provide a foundation for ongoing and new scientific enquiry to more effectively assist families affected by childhood hearing loss and to optimize health services.

## Abbreviations

ASL: American Sign Language; CI: Confidence interval; DSR: Distiller Systematic Review Software; PRISMA: Preferred Reporting Items for Systematic Reviews and Meta-Analyses for Protocols.

## Competing interests

The authors declare that they have no competing interests.

## Authors’ contributions

EMF conceived the project and is the guarantor. EMF, AS, CG, and DM developed the methods. EMF drafted the protocol. All authors reviewed and approved the final version.

## Authors’ information

EMF is Associate Professor in the Audiology and Speech-Language Program, Faculty of Health Sciences at the University of Ottawa and Senior Scientist at the Children’s Hospital of Eastern Ontario Research Institute. AS is Senior Clinical Research Associate, Knowledge Synthesis Group, Centre for Practice-Changing Research, Ottawa Hospital Research Institute. CG is Senior Research Program Manager, Knowledge Synthesis Group, Centre for Practice-Changing Research, Ottawa Hospital Research Institute. DM is Associate Professor, Department of Epidemiology and Community Medicine, Faculty of Medicine, University of Ottawa and Senior Scientist, Clinical Epidemiology, Ottawa Hospital Research Institute.

## Supplementary Material

Additional file 1 MEDLINE Search Strategy. Click here for file
