# Supplementary material for: The effects of sign language on spoken language acquisition in children with hearing loss: a systematic review protocol
Source: Syst Rev. 2013 Dec 6;2:108. doi: 10.1186/2046-4053-2-108 (PMC4029089; doi:10.1186/2046-4053-2-108)
Supplement: Additional file 1 — MEDLINE Search Strategy. [file 2046-4053-2-108-S1.docx]

## Additional file 1: MEDLINE Search Strategy

1. exp Hearing Loss/

2. (hearing adj (loss or impair$ or disorder*)).tw.

3. deaf$.tw.

4. (prelingual$ or pre-lingual$).tw.

5. (sensorineural$ or sensori-neural$).tw.

6. congenital.mp.

7. or/1-6

8. auditory verbal.tw.

9. ((speech or auditory) adj2 feedback).tw.

10. cued speech.tw.

11. (listen* and (spoken or speak*)).tw.

12. oral approach$.tw.

13. aural.tw.

14. Lipreading/

15. (lipread$ or lip read$ or speechread$ or speech read$).tw.

16. or/8-15

17. Manual Communication/ or Sign Language/

18. (sign$ language or sign$ english).tw.

19. ASL.tw.

20. visual language.tw.

21. (baby sign or infant sign).tw.

22. or/17-21

23. Communication Methods, Total/

24. total communication.tw.

25. simultaneous communication.tw.

26. (multilingual or multi-lingual).tw.

27. (bicultural or bi-cultural).tw.

28. (bilingual or bi-lingual).tw.

29. bi bi.tw.

30. or/23-29

31. exp clinical trial/

32. clinical trial.pt. or randomized.ti,ab. or placebo.ti,ab. or randomly.ti,ab. or trial.ti,ab. or groups.ti,ab.

33. ((control$ or clinical or comparative$) adj2 (trial$ or stud$)).mp.

34. exp Epidemiologic studies/ or Case-control studies/ or Retrospective studies/ or Cohort studies/ or Longitudinal studies/ or Cross-sectional studies/

35. between group design$.mp.

36. control group$.mp.

37. (cohort stud$ or longitudinal).mp.

38. (case adj2 (series or control$)).mp.

39. ((consecutive or clinical) adj2 case$).tw.

40. ((control$ or intervention or evaluation or comparative or effectiveness or evaluation or feasibility) adj3 (trial or studies or study or program or design)).tw.

41. or/31-40

42. 7 and ((16 and 22) or 30) and 41

43. 42 and ((Infan* or newborn* or new-born* or perinat* or neonat* or baby or baby* or babies or toddler* or minors or minors* or boy or boys or boyfriend or boyhood or girl* or kid or kids or child or child* or children* or schoolchild* or schoolchild).mp. or school child.ti,ab. or school child*.ti,ab. or (adolescen* or juvenil* or youth* or teen* or under*age* or pubescen*).mp. or exp pediatrics/ or (pediatric* or paediatric* or peadiatric*).mp. or school.ti,ab. or school*.ti,ab. or (prematur* or preterm*).mp.)

44. limit 43 to ("in data review" or in process or "pubmed not medline")

45. 42 and (child* or adolescent or infan*).mp.

46. 44 or 45

47. limit 46 to yr="1985 -Current"
